# A vocal basis for the affective character of musical mode in melody

**DOI:** 10.3389/fpsyg.2013.00464

**Published:** 2013-07-31

**Authors:** Daniel L. Bowling

**Affiliations:** Department of Cognitive Biology, University of ViennaVienna, Austria

**Keywords:** music, emotion, voice, mode, interval-size

## Abstract

Why does major music sound happy and minor music sound sad? The idea that different musical modes are best suited to the expression of different emotions has been prescribed by composers, music theorists, and natural philosophers for millennia. However, the reason we associate musical modes with emotions remains a matter of debate. On one side there is considerable evidence that mode-emotion associations arise through exposure to the conventions of a particular musical culture, suggesting a basis in lifetime learning. On the other, cross-cultural comparisons suggest that the particular associations we make are supported by musical similarities to the prosodic characteristics of the voice in different affective states, indicating a basis in the biology of emotional expression. Here, I review developmental and cross-cultural studies on the affective character of musical modes, concluding that while learning clearly plays a role, the emotional associations we make are (1) not arbitrary, and (2) best understood by also taking into account the physical characteristics and biological purposes of vocalization.

## Introduction

One of the oldest and most prevalent ideas in the history of music is that there is a special connection between music and emotion (Kivy, 2002, 14). Today we know that the emotional coloring of a piece of music results from a complex interplay between acoustical properties, expectation, context, and a listener's personal experience. Efforts to understand the role of acoustical properties have been particularly successful. A great deal is now known about the affective contributions of tempo, intensity, pitch, spectral energy distribution, tone attack, and microstructural irregularity—and a coherent biological framework for understanding why they affect us has been offered in terms of similarity to corresponding properties in vocal affect expression (reviewed in Juslin and Laukka, 2003). The focus here is on the affective contribution of musical *mode*, which has proven a greater challenge to understand. In musicology, the term “mode” is used to refer to a variety of different concepts, only loosely related by their usage in the study of scales and melodies (Randel, 1986, 499). This ambiguity of meaning has generated some confusion, particularly when comparing different musical traditions. Thus, as a first step, I define mode as a set of musical tones and tone-relationships that are used to create a melody. By avoiding additional related concepts such as rules for ornamentation or pre-specified melodic motifs, this simple definition can be applied to music from a wide variety of traditions.

The notion that the mode of a melody influences the perception of its emotional coloring has been a part of music theory since antiquity. In *The Republic*, Plato treats it as common sense that certain modes are best suited for the representation of particular feelings or states of character (Plato, 375 BCE/1955, 93–95). Similarly, in Indian musical traditions, descriptions of which modes are appropriate for various emotions are found in ancient Sanskrit texts, such as the *N*ātyśāstra (~200 CE; Capwell, 1986, 779; Devy, 2002, 3–4). Further examples come from the Middle East and East Asia, where the affective connotations of different modes are documented in Persian and Japanese musical traditions (Nettl, 1986, 531; Hoshino, 1996).

Despite these rich historical and cross-cultural opportunities, the affective character of musical modes has almost exclusively been studied in the context of post-renaissance Western music, where the most salient examples of mode-emotion associations are based on the major and minor modes. When other factors such as tempo and intensity are carefully controlled, music composed using the major mode is typically heard as relatively positive and excited (e.g., joyful), whereas music composed using the minor mode is heard as relatively negative and subdued (e.g., sad; Hevner, 1935; Crowder, 1984, 1985; Kastner and Crowder, 1990; Gerardi and Gerken, 1995; Gregory et al., 1996; Peretz et al., 1998; Dalla Bella et al., 2001; Gagnon and Peretz, 2003). These connotations appear to have persisted for over 400 years—the 16th century Italian music theorist and composer Gioseffo Zarlino similarly described the effect of the major mode as “gay and lively,” and the minor mode as “sad and languid” (Zarlino, 1558/1968, 21–22).

The historical, cross-cultural, and repeatedly verified relationships between musical modes and emotions make it clear that these associations must be addressed by any theory of musical emotions. In spite of this, most modern theories downplay mode, relegating it to a catch-all of cultural convention, and in so doing pay little attention to the possibility of biological roots. In what follows, I advance a biological approach to mode-emotion associations by: (1) demonstrating the logical error of using evidence that mode-emotion associations are learned, as evidence against biological underpinnings; (2) presenting the evidence that mode-emotion associations exhibit acoustical similarities across cultures; and (3) extending the biological framework of vocal imitation proposed by Spencer (1857, 1890) to account for why we associate modes and emotions.

## The evidence for learning

Most modern accounts of why music expresses emotion suggest that emotional associations with mode are learned through exposure to the conventions of a particular musical culture (e.g., Lundin, 1967, 166–169; Juslin and Laukka, 2003; see also Huron, 2008; Trainor and Corrigall, 2010). This position is supported by evidence that mode-emotion associations develop with age, and are more reliably made by individuals with musical training. One experimental paradigm involves presenting children of different ages with major and minor music and asking them to “match the feeling” by selecting among schematic drawings of various facial expressions (Dolgin and Adelson, 1990). Using this approach, a number of studies have found that culturally appropriate mode-emotion associations do not develop until 6–8 years of age, before which they are not reliably made (Gerardi and Gerken, 1995; Gregory et al., 1996; Dalla Bella et al., 2001; but see Kastner and Crowder, 1990 for emergence at age 3). The perception of major and minor chords has also been studied in infancy (6 months) using a preferential looking paradigm (Crowder et al., 1991). In accord with the results for young children, no reliable preferences were observed for major over minor (or vice versa). With respect to musical training, a number of studies have shown that individuals who have had explicit instruction in Western music make culturally appropriate mode-emotion associations more reliably than those who have not (e.g., Heinlein, 1928; Hevner, 1935). Importantly however, these studies also show that musical training is not necessary for appropriate associations to be made (see also Dalla Bella et al., 2001). These findings, together with the fact that mode-emotion associations are well established in adulthood (Hevner, 1935; Crowder, 1984, 1985; Gerardi and Gerken, 1995; Gregory et al., 1996; Peretz et al., 1998; Dalla Bella et al., 2001; Gagnon and Peretz, 2003), suggest a pattern of learning over the course of development.

Evidence that mode-emotion associations strengthen with age and musical training is often taken to imply that such associations are arbitrary, arising solely through learning the conventions of a given musical culture (Heinlein, 1928; Lundin, 1967, 166–169; Gregory et al., 1996). Over the past 70 years, however, ethologists have repeatedly demonstrated that evidence for learning does not necessarily imply an absence of biological preparation. On the contrary, learned associations are often supported by inherited predispositions. For example, honeybees must learn to associate specific flower types with food, but they instinctively attend flowerlike objects (Gould and Marler, 1987). Similarly, rats must learn whether specific foods are safe for consumption, but they are predisposed to attend olfactory as opposed visual or auditory cues in doing so (Garcia and Koelling, 1966). Another example comes from songbirds that despite having to learn local song dialects from adult conspecifics, are predisposed to recognize and preferentially learn species-typical song patterns (Marler, 1991). Among primates, young Vervet monkeys must learn to emit “eagle” alarm calls to objects that pose an aerial threat (such as eagles or hawks), but are predisposed to emit these calls to a wide-range of objects moving overhead, including for example, falling leaves (Seyfarth et al., 1980). Human children must also learn about potential sources of danger, a process that is in part supported by perceptual biases to associate certain classes of animals (e.g., spiders or snakes) with fear responses (Lobue and DeLoache, 2008). Finally, and perhaps of even greater significance, we are somehow predisposed to learn the myriad associations and rules that constitute language (Tomasello, 1999; Chomsky, 2006). In sum, these examples underscore the importance of biology in explaining perception and behavior and caution against the assumption that evidence for learning in mode-emotion associations precludes a species-wide predisposition to make them.

## Mode-emotion associations across cultures

Interpretation of developmental studies is complicated by the confounding roles of perceptual and cognitive development. Thus, in the developmental studies alluded to above, it remains possible that mode-emotion associations are somehow made by young children but were not observed due to perceptual or cognitive limitations, such as difficulties with infering harmonic structure (see Gerardi and Gerken, 1995) or because the task of pointing to schematic faces representing emotional reactions to music requires cognitive skills poorly developed at the ages examined (see Dalla Bella et al., 2001). Cross-cultural studies offer a clear advantage here because adults from different cultures presumably have comparable perceptual and cognitive abilities. Accordingly, a number of studies have examined how adults respond to emotion in music from unfamiliar cultures. Few of these studies however, have been specifically designed to assess the influence of mode, which is thus typically confounded with other variables such as tempo and intensity. Nevertheless, this body of work shows that adult listeners perceive the emotions intended by composers/performers in culturally unfamiliar music with considerable accuracy (particularly for joy and sadness), and that their emotional judgments vary with mode in a way that is either in agreement with unfamiliar traditions (Balkwill and Thompson, 1999; Balkwill et al., 2004), or at least matches the judgments of listeners native to the culture in question (Fritz et al., 2009; Zacharopoulou and Kyriakidou, 2009). Only one cross-cultural study has isolated the influence of mode on perceived emotion. Hoshino (1996) played simple major and minor melodies to Japanese adults who grew up before WWII and were reportedly unfamiliar with Western musical conventions. Although Hoshino's methods were somewhat unorthodox—subjects were asked to associate melodies with colors, which they later described with emotional labels—she found some evidence for cross-cultural similarity in modal perception: major melodies were described as bright and warm, whereas minor melodies were described as dark and melancholic.

A different approach to studying mode-emotion associations is to compare the structure of music composed using modes from different cultures that are associated with similar emotions. Features that are held in common are candidates for mediating emotional associations. Accordingly, Bowling et al. (2012) compiled melodies composed in modes associated with either negative-subdued or positive-excited emotions in classical South Indian (*Carnatic*) and classical Western music and compared their structure in terms of the sizes of the intervals that occur between adjacent melody notes (see also Huron, 2008). In both traditions, it is apparent that the use of modes associated with negative-subdued emotion results in melodies with a significantly greater proportion of smaller intervals (<200 cents^1^), whereas the use of modes associated with positive-excited emotion results in melodies with a significantly greater proportion of larger intervals (≥200 cents). These results are not specific to classical music, as the same pattern has also been observed in major and minor Finnish Folk melodies (Bowling et al., 2010). These comparisons cast further doubt on the idea that the associations between mode and emotion are inherently arbitrary.

## A vocal basis for the affective character of musical modes

What are cross-cultural similarities in mode-emotion associations based on? In his essay “On the Origin and Function of Music,” Herbert Spencer (1857) proposed that music expresses emotion by imitation and exaggeration of acoustical properties of emotional expression in the voice. His logic entails two steps. First, he argued that the physiological components of emotion affect the mechanisms of vocal production and thus the acoustical properties of the voice, resulting in routine associations between those properties and the subjective components of emotional experience. And second, he argued that by employing the same acoustical properties, music gains access to the same emotional associations. Although Spencer's theory co-exists with several others that attempt to explain how music conveys emotion [reviewed in Davies (2001); see also Crowder (1984) and Juslin and Västfjäll (2008)], increasing evidence of acoustical similarities between music and voice, paired with careful consideration of the various relationships that might explain them (Juslin and Laukka, 2003), have provided strong support for Spencer's vocal imitation hypothesis.

While Spencer (1857, 399) did not specifically consider musical mode, he did comment on interval size, noting that “calm speech is comparatively monotonous” whereas “emotion makes use of fifths, octaves, and even wider intervals.” Distinguishing between different types of emotion, more recent studies have found that frequency contours in sad speech are relatively flat and stable, whereas frequency contours in joyful speech are more dynamic and variable [reviewed in Scherer (1986, 2003)]. With respect to interval-size, Bowling et al. (2012) calculated the frequency differences between adjacent voiced intensity maxima in recordings of sad and joyful speech. In parallel with the pattern of interval-sizes found in modal melodies (see above), sad speech comprised a greater proportion of smaller intervals, while joyful speech comprised a greater proportion of larger intervals. Furthermore, the interval-size at which the reversal in prevalence occurred between the two emotional conditions was roughly the same in speech and music, between 100 and 200 cents.

Evidence of similarities in interval-size between musical and vocal expression have also been found by Curtis and Bharucha (2010), who report that descending minor thirds (-300 cents) frequently occur between the first and second syllables of two-syllable expressions conveying sadness (e.g., “come on”). This result is of particular interest because minor thirds play a central role in distinguishing music composed in the minor modes (Bowling et al., 2010). Accordingly, Curtis and Bharucha argue for the possibility of an “interval code” by which the occurrence of specific intervals signal specific emotions in music and the voice. Two issues complicate this interpretation. First, only descending minor thirds ending on the tonic (the “tonal center” of a mode) are emphasized in minor vs. major music. Defined more generally (i.e., not limited to those intervals ending on the tonic), descending minor thirds are only marginally more prevalent in minor vs. major melodies: by 0.1% in classical Western music and 0.4% in Finnish folk music (Bowling et al., 2010, 2012). Furthermore, the opposite pattern occurs in Carnatic melodies, where descending minor thirds are actually more prevalent in melodies composed in modes associated with joy (accounting for 8% of melodic intervals) than they are in melodies composed in modes associated with sadness (accounting for 4.2%; Bowling et al., 2012). It remains possible that tonic minor thirds tap into some form of interval code, but further research is needed to explore whether or not the concept of a tonic has any bearing in speech. Second, there is conflicting evidence as to whether or not specific musical intervals are emphasized in speech prosody. In contrast to Curtis and Bharucha, Bowling et al. (2012) found no evidence of emphasis at descending minor thirds in two-syllable expressions of sadness. Methodological differences in speaker selection, method of interval calculation, and histogram-bin size are likely sources of this difference. However, aside from these conflicting findings over the importance of relative vs. specific interval-sizes in mode-emotion associations, the available evidence clearly indicates that interval-size, like many other acoustical properties, varies with emotion in music and the voice in a parallel fashion.

## Theoretical accounts

Five theoretical frameworks can account for the relationship between interval-size in modal music and vocal expression. In what follows, I argue for the exclusion of four of them in favor of the fifth, i.e., Spencer's theory that music expresses emotion by imitating the voice. Juslin and Laukka took a similar approach in their 2003 review of acoustical similarities between musical and vocal expression, although they did not explicitly consider interval-size.

### Explanation 1 - interval-sizes in musical and vocal expression are entirely unrelated, observed similarities are coincidental

The principal argument against this possibility is that the same relationship between interval-size and emotion is observed in music and speech from different musical traditions and cultures. Whereas we can be fairly certain that basic acoustical properties of vocal expression are conserved across cultures (Elfenbein and Ambady, 2002), assessments of interval-size in mode-emotion associations from additional musical traditions could potentially strengthen the argument against spurious correlation (Persian music offers perhaps the best opportunity here). If such assessments are undertaken in the future, the prediction from the Carnatic and Western music comparisons (see above) is that musical traditions that systematically associate smaller intervals with positive-excited emotion, and/or larger intervals with negative-subdued emotion, are either non-existent or exceptionally rare. Additionally, the creation of new tonal traditions with these “backward” emotional associations should be considerably more difficult than the creation of new tonal traditions with “normal” associations.

A second argument against a coincidental interpretation of interval-size similarities in musical and vocal expression is that interval-size is part of a larger pattern of overall acoustic similarity between vocal and musical expression that includes a host of other properties. The possibility that all of these similarities are coincidental is remote (Juslin and Laukka, 2003), and there is no apparent reason to make an exception of interval-size.

### Explanation 2—interval-sizes in musical and vocal expression are related, but only through the common influence of a third causal factor

The arguments against this explanation are best made using examples. Thus, one potential third factor that could influence music and the voice is the expression of emotion through body postures and movements (Kivy, 2002, 37–48). According to this hypothesis, acoustical properties such as tempo or intensity, express emotion in both music and the voice because they imitate analogous properties of bodily expression, such as the speed and force of movements. However, it is unclear how this hypothesis would apply to the sizes of frequency intervals, as there is no clear analogue in body postures or movements. This problem reflects a general limitation of *third causal factor* accounts of interval-size similarities: frequency intervals are essentially unique to music and the voice. It is true that a metaphorical similarity between frequency intervals in music and the size/extent of body movements could be drawn, but it is unclear why this would be more plausible than a direct similarity with frequency intervals in the voice.

Another type of third causal factor interpretation considers influence apart from explicit imitation. For example, it is possible that a physiological factor such as arousal determines the relationship between emotion and interval-sizes in music and the voice. With respect to the voice one could postulate that changes in arousal determine interval-size by regulating the energy available to the muscles that control breathing and laryngeal posture. With respect to music however, this link between arousal and interval-size does not necessarily apply. The reason is that the forms of musical instruments are not physiologically constrained. Accordingly, the relationship between muscular effort and interval-size may be decoupled (as it is to some extent with a piano or guitar, and to a fuller extent with computer software used for composition) or even reversed (imagine an instrument on which the more forcefully one plays, the slower, softer, and smaller frequency intervals become). The lack of necessary connection between arousal and interval-size in instrumental music poses a problem for an arousal-based third causal factor account because it makes clear that arousal does not always determine interval-sizes in music. This example thus provides another general argument against third causal factor accounts of interval-size similarities in musical and vocal expression. For any cause that does not explicitly comprise frequency intervals, a necessary link to interval-size must be demonstrated in both domains.

### Explanations 3–5

Each of the remaining explanations posits some form of causal relationship between interval-size in music and the voice. Before considering them, it is necessary to draw a distinction between vocal and non-vocal music since any argument claiming that emotional expression in the voice derives from vocal music (or vice versa) is inherently circular. Keeping this in mind, there are three possible causal relationships that could exist between interval-size in musical and vocal expression: (3) *the voice imitates music*; (4) *both music and the voice imitate each other, with neither being primary*; and (5) *Spencer*'*s theory that music imitates the voice*.

The critical evidence for deciding between these alternatives concerns the evolutionary primacy of affective vocal expression. Despite the well-known debate between Spencer and Darwin over whether music derives from speech or speech derives from music, both men agreed on the primacy of some form of vocalization in auditory affective communication (Darwin, 1879/2004, 638; 1889/1998, 88–99; Spencer, 1857, 1890), and the evidence has only grown stronger since. With respect to musical instruments the earliest uncontested archaeological evidence—a pair of flutes made from the wing bones of a swan—dates to approximately 37000 years ago (reviewed in Fitch, 2006). In contrast, many of the neural and physiological mechanisms responsible for vocal affect expression (e.g., descending motor control from the brainstem, and various aspects of laryngeal anatomy) are shared by a large variety of mammals, suggesting relatively ancient phylogenetic roots (Jürgens, 1992; Ploog, 1992).

Even if one pushes the origins of instrumental music back to our common ancestor with *H. neaderthalensis* (as suggested by certain interpretations of slightly older flute-like objects found with other Neaderthal artifacts; Kunej and Turk, 2000), or even further back to our last common ancestor with chimpanzees and gorillas, (which both display some forms of drumming behavior; Fitch, 2006), the primacy of vocal affect expression remains incontrovertible. Explanations that the voice imitates music, or that both imitate each other with neither having primacy (explanations 3 and 4 above), are both incompatible with these facts. Only Spencer's theory, that interval-size in music expresses emotion by imitation and exaggeration of interval-size in the voice (explanation 5 above), is compatible with current archeological and phylogenic data relevant to the ages of vocal affect expression and instrumental music.

## A note about harmony

The present discussion has focused on changes in frequency over time (i.e. melody), but the affective character of musical modes is also realized in the simultaneous presentations of multiple frequencies, i.e., harmonies or chords (Heinlein, 1928; Crowder, 1984, 1985). Nevertheless, the relationship between melodic interval-size and emotion discussed here may also have relevance for the affective character of mode in harmony. Krumhansl (1990) provides extensive evidence that the notes in a melody line are perceived in the harmonic context of previously occurring notes, even though there is no physical simultaneity. Accordingly, when this context is made explicit in the form of chords, simultaneous intervals may in some sense be interpreted in relation to their melodic counterparts, i.e., the response of the nervous system may be partially redundant between these situations, activating associations originally made in melodic context in harmonic context and vice versa. For alternative theories on the affective impact of mode in harmony, based on sensory dissonance, familiarity with harmonic spectra, or sound symbolism, see Helmholtz (1877/1895) (211–219), Crowder (1984), and Cook (2007), respectively.

## Conclusion

Explanations of mode-emotion associations that rely solely on lifetime learning of arbitrary cultural conventions are incompatible with evidence suggesting that the particular associations we make are similar across cultures and constrained by the interval-size properties of modes as expressed in melody. The parallel patterns observed between interval-size and emotion in modal music and the voice suggest a relationship between these domains. Upon consideration of the possible explanations that could account for interval-size similarities between musical and vocal expression, Spencer's theory that music imitates the voice is the only explanation that is entirely consistent with the available evidence. Accordingly, it seems reasonable to conclude that the affective character of modes as realized in melody is—like many other aspects of music—best understood by also taking into account the physical characteristics and biological purposes of vocalization.

## Financial disclosure

This work was supported by a grant from the National Science Foundation [BCS-0924181] awarded to Dale Purves, and a European Research Council advanced grant [No. 230604 “SOMACCA”] awarded to Tecumseh Fitch.

### Conflict of interest statement

The author declares that the research was conducted in the absence of any commercial or financial relationships that could be construed as a potential conflict of interest.

## Footnotes

^1^Cents are units of frequency interval size. One cent is 1/100 of an equally tempered semitone. An octave thus comprises 1200 cents. The formula for calculating the size of an interval between two frequencies (F1 and F2) in cents C, is C = 1200 × log_2_(F1/F2).
